# Effect of Buzhong Yiqi decoction on anti-acetylcholine receptor antibody and clinical status in juvenile ocular myasthenia gravis

**DOI:** 10.1097/MD.0000000000027688

**Published:** 2021-11-05

**Authors:** Jiaduo Li, Guoyan Qi, Yaling Liu

**Affiliations:** a Center of Treatment of Myasthenia Gravis, People‘s Hospital of Shijiazhuang Affiliated to Hebei Medical University, China; b Gastroenterology Department, People‘s Hospital of Shijiazhuang Affiliated to Hebei Medical University, China.

**Keywords:** acetylcholine receptor, decoction, juvenile, myasthenia gravis, ocular

## Abstract

Ocular myasthenia gravis (MG) is the mainly widespread type of MG among juveniles. Buzhong Yiqi decoction (BZ) is a well-known traditional Chinese medicine prescription for treating MG. It has rarely been discussed whether the concentration of anti-acetylcholine receptor (AChR) antibodies is related to the clinical status of juvenile ocular myasthenia gravis (JOMG) treated with BZ.

The patients with JOMG who had more than once AChR-antibody (ab) test and treated with BZ were retrospectively identified from June 2013 to January 2020 in the first hospital in Shijiazhuang. The presence or absence of ocular symptoms was used to grade the effectiveness of treatment. Generalized estimating equations logistic regression analysis was used to evaluate the effect of AChR ab concentration on the clinical status of MG.

A total of 549 AChR-ab tests were performed in 135 patients, and the corresponding clinical status was recorded. One hundred two patients received treatment with BZ only and 33 combined with immunosuppressive drugs. In the group receiving only BZ treatment, the anti-acetylcholine receptor ab concentration was positively correlated with the clinical score after treatment.

The results suggest that BZ could affect the AChR-ab. Repeated AChR-ab assays can provide information about the clinical status. For JOMG patients who only receive Buzhong Yiqi Decoction treatment, this can support treatment decisions.

## 1 Introduction

Juvenile ocular myasthenia gravis (JOMG) is an autoimmune disease with ocular muscle involvement as the main symptom and affecting the postsynaptic membrane of neuromuscular junction.^[1,2]^ Ptosis and diplopia are common symptoms.^[3,4]^ Juveniles are at the peak of the disease in China and Japan, and a large proportion of them develop ocular symptoms before the age of 14.^[5–7]^ Prednisone is considered to be the first-line drug for the treatment of JOMG.^[8]^ Fear of side effects or ineffective treatment has led some parents and juveniles to reject or give up prednisone treatment. Buzhong Yiqi decoction (BZ) is a safe and effective traditional Chinese medicine for the treatment of myasthenia gravis (MG).^[9,10]^ Although some JOMG patients in Chinese hospitals have been treated with Buzhong Yiqi decoction for many years, there is still a lack of a prognostic marker to support the treatment decision of BZ. Anti-acetylcholine receptor (AChR) antibodies can be detected in 80% of MG patients.^[2,11]^ Repeated tests of the AChR-antibody (ab) can provide information on the clinical progress of immunosuppressive therapy in patients with MG.^[12]^ Therefore, ab testing has a potential correlation in patient follow-up, which may contribute to future management and treatment decisions of BZ.^[13]^ The purpose of this study was to determine whether there was a correlation between individual AChR-ab concentration and clinical status in patients treated with BZ.

## 2 Methods

### 2.1 Patients selection

Patients under 18 years of age diagnosed with JOMG from June 2013 to January 2020 in First Hospital of Shijiazhuang, which is a MG diagnosis and treatment center, were retrospectively reviewed by medical records. The study was approved by the Ethical Committee of First Hospital of Shijiazhuang. Ocular myasthenia gravis (OMG) was diagnosed according to the Myasthenia Gravis Foundation of America (MGFA) clinical classification,^[14]^ defined as the presence of only ocular muscle weakness or eye closure weakness plus positive test results for at least one of the following assessments: responsive to Tensilon test or anti-cholinergic regimen, decremental response to the 3 Hz repetitive stimulation test, and presence of serum anti-acetylcholine receptor antibodies (≥0.5 nanomoles per liter [nmol/L]). Two neurologists made the diagnosis together. The inclusion criteria included: Juveniles <18 years of age, met OMG diagnostic criteria, untreated or treated with prednisone before BZ treatment. The exclusion criteria included: AChR-antibodies <0.5 nmol/L before BZ treatment, aggravate to generalized MG, No AChR-ab test was performed after BZ treatment.

### 2.2 AChR-ab test

AChR-ab tests for patients were implemented in the clinical laboratory. According to the instructions of the RSR manufacturer in the United Kingdom (http://www.rsrltd.com), the collected serum samples were tested for AChR-antibodies using radioimmunoassay. Positive AChR-ab is defined as a sample concentration of ≥0.5 nmol/L.^[15]^ We received the test results from the central database.

### 2.3 Data used in the study

Data concerning name, sex, date of birth, date of onset, clinical symptoms, diagnosis date, pharmacological history treatment, date of sample test, clinical status, and medication at the time of test, date of BZ treatment were collected. Our sample contains 135 patients in the included period were AChR-ab positive before BZ treatment combined with an ocular symptom. Seventy-three patients without any treatment outside the hospital were only treated with Buzhong Yiqi decoction (BZ-only group), whereas 29 patients who experienced severe side effects and did not achieve satisfactory therapeutic effects of oral prednisone were treated with Buzhong Yiqi decoction instead (BZ-instead group). Besides, 33 patients were treated with Buzhong Yiqi decoction combined with prednisone (BZ-prednisone group).

### 2.4 JOMG clinical evaluation

The presence or absence of ocular symptoms was used to assess the clinical score of the patients. Patients in this study with symptoms were scored as 1 and without symptoms were scored as 0. Then the outcomes of all the AChR-ab tests were associated with the matching JOMG-score.

### 2.5 Statistical analysis

AChR-ab measurements and JOMG score assessments were done at fluctuating time intervals for each JOMG patient. By assuming equivalent correlation structures (intra-individual analysis), corrected repeated measures are used in the same individual. Minimum, maximum, median, mean and standard deviation are used for descriptive statistics. The treatment time is divided into 4 intervals, according to the quartile. Generalized estimating equations model with robust standard errors was used to performs a linear regression (link of identity) of AChR-ab on therapy time, group, and JOMG score with an assumed exchangeable correlation structure within the patients. The generalized estimating equation logistic model was used to analyze the effects of BZ treatment time and ab concentration on the JOMG-score.^[16]^ We further measured the impact of different groups and the level of the AChR-ab on the JOMG score over BZ treatment time. The results are reported in the form of odds ratio (OR) and 95% confidence interval. SPSS 21 and STATA were used for statistical analyses.

## 3 Results

Five hundred forty-nine AChR-ab tests were performed in 135 JOMG patients in total, and the corresponding clinical status was recorded. Eighty-three female and fifty-two male patients were included, with an average age of 4.8 years (10 months-16.5 years). The average number of AChR-ab tests was 4.1 (2-9). The mean serum AChR-ab concentration before BZ treatment was 2.32 nmol/L (0.5-9.4). The average duration of onset before BZ treatment was 1.3 years (0-12 years), and the average follow-up time after BZ treatment was 1.3 years (0.1-5.7 years). In the BZ-only group, there were 32 female and 41 male patients with an average age of 4.3 years. The average concentration of serum AChR-ab before BZ treatment was 1.58 nmol/L. The interval between the onset of JOMG and BZ treatment was 0.61 years. In the BZ-Prednison group, there were 20 female and 13 male patients with an average age of 4.8 years. The average concentration of serum AChR-ab before BZ treatment was 1.36 nmol/L. The interval between the onset of JOMG and BZ treatment was 1.41 years. In the BZ-instead group, there were 22 female and 7 male patients with an average age of 5.8 years. The average concentration of serum AChR-ab before BZ treatment was 2.36 nmol/L. The interval between the onset of JOMG and BZ treatment was 2.62 years. Statistically significant differences between differentially treated groups are indicated in the interval between the onset of JOMG and BZ treatment, *P* < .05 (Tables 1 and 2).

**Table 1:** Baseline characteristics at first antibody test of all myasthenia gravis patients (n = 135).

Variables	BZ-only (73)	BZ-Prednison (33)	BZ-instead (29)	*P* value
Age, mean (SD) (yrs)	4.3 (3.6)	4.8 (3.5)	5.8 (3.3)	.154^∗^
Gender, n (%)				.181^†^
Female	41 (56.2)	20 (60.6)	22 (75.9)	
Male	32 (43.8)	13 (39.4)	7 (24.1)	
AChR-ab concentration, median (IQR)(nmol/L)	1.58 (2.24)	1.36 (2.67)	2.36 (2.79)	.798^∗^
Age at MG onset (yrs)	3.67 (3.23)	3.35 (2.63)	3.14 (2.21)	.68^∗^
Interval between disease onset and initiate BZ (yrs)	0.61 (1.74)	1.41 (2.10)	2.62 (2.77)	.001^∗^
History of prior treatment	Nona	Prednisolone	Prednisolone	

ab = antibody, AChR = acetylcholine receptor, BZ = Buzhong Yiqi decoction, IQR = interquartile range, MG = Myasthenia gravis, SD = standard deviation.

∗ Analysis of variance.

† Exact chi-square test.

**Table 2:** Distribution of anti-acetylcholine receptor antibody tests.

Number of tests	Number of patients
2	35
3	19
4	28
5	29
6	11
7	8
8	2
9	3

The 4 intervals of BZ treatment time was 0 to 0.25 year (0-3 months), 0.25 to 0.58 years (3-7 months), 0.58 to 1 year (7-12 months), and the rest (12 months-5.67 years). One hundred thirty, ninety-seven, eighty-four, and one hundred three AChR-ab tests were carried out at these 4 intervals, respectively. The overall mean JOMG-score decreased from 0.9 to 0.2, while AChR-ab concentration decreased from 2.33 to 1.21 nmol/L as the treatment time increased (Table 3). With each increase of BZ treatment time of 1 interval, there was an associated decrease in average AChR-ab of about 0.19 nmol/L. With 1 increased of the JOMG score, there was an associated increase in average AChR-ab of about 0.69 nmol/L, as exhibited in Table 4.

**Table 3:** Descriptive statistics for myasthenia gravis score (0-1) and anti-acetylcholine receptor antibody concentration (nmol/L).

		MG-score					AChR-antibody concentration				
Time^∗^	No.	Median	Mean	SD	Min	Max	Median	Mean	SD	Min	Max
Before BZ	135	1	0.9	0.3	0	1	1.55	2.33	1.92	0.5	9.4
0-3 mo	130	1	0.6	0.5	0	1	1.86	2.28	1.92	0.01	8.18
3-7 mo	97	0	0.3	0.5	0	1	1.08	1.54	1.68	0.01	6.32
7-12 mo	84	0	0.2	0.4	0	1	0.74	1.35	1.49	0.01	5.77
12-68 mo	103	0	0.3	0.5	0	1	0.52	1.21	1.55	0.01	6.62

AChR = anti-acetylcholine receptor, BZ = Buzhong Yiqi decoction, MG = myasthenia gravis, No. = number, SD = standard deviation.

∗ 1st quartile: 0-3 mo; 2nd quartile: 3-7 mo; 3rd quartile: 7-12 mo; 4th quartile: 12 mo-68 mo.

**Table 4:** The effects of juvenile ocular myasthenia gravis score (0-1), treatment time (mo) and group on anti-acetylcholine receptor antibody (nmol/L).

AChR-antibody	Coef.	Std. err.	z	*P*>|z|	[95% conf. interval]	
Treatment time	–0.1898567	0.0529193	–3.59	.000	–0.2935766	–0.0861368
Group	0.187442	0.1644233	1.14	.254	–0.1348218	0.5097059
JOMG score	0.6686138	0.1030997	6.49	.000	0.4665421	0.8706855
_Cons	1.655803	0.3410855	4.85	.000	0.9872874	2.324318

Wald chi^2^ (3) = 108.44; Prob > chi^2^ = .0000. AChR = anti-acetylcholine receptor, Coef. = coefficient, JOMG = juvenile ocular myasthenia gravis score, Std. err. = standard error.

The estimated odds ratio for AChR-ab was about 1.4, with the interpretation that the odds of a JOMG score as 1 increased by 40% with each nmol/L grew in AChR-ab. The estimated odds ratio for treatment time was about 0.47, with the interpretation that the odds of a JOMG score as 1 decreased by 53% with each increased in treatment interval. The above results were similar in all patients and groups of patients. Tables 5 and 6 display the results.

**Table 5:** The effects of anti-acetylcholine receptor antibody (nmol/L), treatment time (mo) and group on juvenile ocular myasthenia gravis score (0-1).

JOMG-score	Coef.	Std. err.	z	*P*>|z|	[95% conf. interval]	
AChR-antibody	1.398973	0.1243215	3.78	.000	1.175347	1.665146
Treatment time	0.4661513	0.044437	–8.01	.000	0.3867088	0.5619138
Group	0.9272061	0.1555725	–0.45	.652	0.6673541	1.288238
_Cons	6.297531	2.216395	5.23	.000	3.159314	12.55301

Wald chi^2^ (3) = 108.44; Prob > chi^2^ = .0000. AChR = anti-acetylcholine receptor, Coef. = coefficient, JOMG = juvenile ocular myasthenia gravis score, Std. err. = standard error.

**Table 6:** The effects of anti-acetylcholine receptor antibody (nmol/L) and treatment time (mo) on juvenile ocular myasthenia gravis score (0-1) by group.

JOMG-score	Coef.	Std. err.	z	*P*>|z|	[95% conf. interval]	
BZ-only group^∗^						
AChR-antibody	1.412813	0.2075739	2.35	.019	1.059313	1.884279
Treatment time	0.4002334	0.0635234	–5.77	.000	0.2932338	0.5462766
_Cons	9.682181	3.975526	5.53	.000	4.3298	21.65103
BZ-instead group^†^						
AChR-antibody	1.476053	0.2827234	2.03	.042	1.014058	2.148529
Treatment time	0.4638466	0.0848107	–4.20	.000	0.3241446	0.6637582
_Cons	6.356616	2.870175	4.10	.000	2.623534	15.40158
BZ-Prednison^‡^						
AChR-antibody	1.289943	0.1530525	2.15	.032	1.022292	1.627669
Treatment time	0.5533719	0.0995817	–3.29	.001	0.3889015	0.7873985
_Cons	2.350253	1.312963	1.53	.126	0.7863809	7.024836

AChR = anti-acetylcholine receptor, BZ = Buzhong Yiqi decoction, Coef. = coefficient, JOMG = juvenile ocular myasthenia gravis, Std. err. = standard error.

∗ Patients treated with BZ only.

† Patients treated with BZ instead Prednison.

‡ Patients treated with BZ and Prednison.

Table 7 also gives an overall test of whether the JOMG score varies with treatment time, group, and AChR-ab. The time intervals after all treatments were statistically different from those before BZ treatment. This shows that the concentration of AChR ab has an effect on the JOMG score during the whole study period. Although the impact of groups on the JOMG score was not statistically significant, there was a statistical difference between the BZ-only and BZ-Prednison group, but no statistical difference between the BZ-only and BZ-instead group.

**Table 7:** The effects of anti-acetylcholine receptor antibody (nmol/L), treatment time (mo) and group on juvenile ocular myasthenia gravis score (0-1).

JOMG-score	Coef.	Std. err.	z	*P*>|z|	[95% conf. interval]	
0-3 mo	0.1290985	0.0467159	–5.66	.000	0.063519	0.262385
3-7 mo	0.0439733	0.016618	–8.27	.000	0.0209658	0.0922289
7-12 mo	0.0224996	0.0092087	–9.27	.000	0.0100877	0.0501831
12-68 mo	0.0392968	0.0151449	–8.40	.000	0.0184631	0.083639
BZ-instead group^†^	1.010358	0.3511385	0.03	.976	0.5112695	1.996645
BZ-Prednison group^‡^	0.4874544	0.157574	–2.22	.026	0.2586875	0.9185283
AChR-antibody	1.344579	0.1014235	3.93	.000	1.159789	1.558812
_Cons	9.337715	3.61751	5.77	.000	4.369964	19.95278

Wald chi^2^ (7) = 139.91; Prob > chi^2^ = .0000. AChR = anti-acetylcholine receptor, BZ = Buzhong Yiqi decoction, Coef. = coefficient, JOMG = juvenile ocular myasthenia gravis, Std. err. = standard error.

† Patients treated with BZ instead Prednison.

‡ Patients treated with BZ and Prednison.

## 4 Discussion

The concentration of the AChR-antibodies in patients with JOMG treated with Buzhong Yiqi decoction was positively correlated with the JOMG score. It is suggested that Buzhong Yiqi decoction can reduce the concentration of AChR-antibodies and improve the clinical state at the same time. The clinical status of patients with JOMG improved over time in this study, consistent with previous studies.^[9,10]^ The clinical status of patients with JOMG treated with Buzhong Yiqi decoction can be predicted by repeated determination of AChR-ab concentration. As a valid biomarker, the AChR-ab can reveal the reaction degree of Buzhong Yiqi decoction and help clinicians to modify or retain the treatment of Buzhong Yiqi decoction. The subjective symptoms and objective signs of patients with MG are very complex. It is challenging to evaluate and compare the clinical status of patients with multiple MG types at the same time. JOMG patients with positive AChR-ab were selected as the object of our study, which simplified the complexity of scoring.

We analyzed the effects of AChR-ab, treatment time and group on JOMG score, the results are shown in Table 7. The BZ-only group was used as the control group. The statistical results showed that there was a difference in the effect on JOMG score between the BZ-Prednisone group and the BZ-only group. Compared with the BZ-only group, the BZ-Prednisone therapy reduced the probability of JOMG score 1. The 2 groups should be treated differently in terms of the frequency and concentration of AChR-antibodies detection. However, there was no difference in the effect on JOMG score between the BZ-instead group and the BZ-only group. This suggests that for patients treated with BZ alone, the previous use of prednisone therapy will not affect the JOMG score. Corticosteroids are widely used as first-line immunosuppressants in the treatment of MG. Many serious side effects of long-term steroid use, such as Cushing syndrome, osteoporosis, weight gain, hyperglycemia, hypertension, gastritis or ulcers, anxiety/depression/insomnia (steroid psychosis), no vascular necrosis of joints.^[17]^ There are insufficient studies on the pharmacological effects of Buzhong Yiqi decoction in the treatment of MG and need to be further studied.^[18]^ Buzhong Yiqi decoction has few adverse reactions, and the main adverse reactions are gastrointestinal reactions, which can be improved after symptomatic treatment.^[19]^ In China, more than half of MG patients are juveniles, and not all youths respond well to corticosteroids.^[8]^ Liu et al^[20]^ reported that oral tacrolimus alone could improve the symptoms of children with MG who are ineffective to prednisone treatment. Still, the safety and efficacy of long-term use need to be further confirmed.

STROBE guidelines were followed in the reporting of the study.^[21]^ There were several limitations in the retrospective study. The main limitation is that the study was a retrospective study with no placebo control for comparison, and selection bias could not be ruled out. The timing of AChR-ab testing varies from patient to patient, which may have an impact on the interpretation of the results. Only patients who have undergone more than once AChR-ab concentration tests are involved in this study, which may have a selection bias. Some patients were in stable condition after treatment with Buzhong Yiqi decoction, so they did not test the AChR-ab again or had an unstable state and changed to other therapies. The follow-up time for some patients is too short. The JOMG score based on more robust and longer-term clinical observation can better reflect the therapeutic effect of Buzhong Yiqi decoction.

## 5 Conclusions

In summary, this study observed that there was a correlation between the decrease of AChR-ab concentration and the improvement of clinical status in JOMG patients treated with Buzhong Yiqi decoction. Repeated determination of the AChR-ab can help monitor the response of JOMG patients to Buzhong Yiqi decoction treatment, thus supporting clinical decision-making.

## Acknowledgments

The authors would like to thank all patients and their families for their support of this research.

## Author contributions

GQ and JL were responsible for the study design. JL and YL analyzed the data and drafted the manuscript. YL and JL contributed to the data collection. GQ, JL, and YL critically reviewed the manuscript and contributed intellectual content. All authors read and approved the final manuscript.

**Data curation:** Yaling Liu.

**Investigation:** Yaling Liu.

**Writing – original draft:** Jiaduo Li.

**Writing – review & editing:** Guoyan Qi.
